# Supplementary figures and images for: Longitudinal Patterns in the Isolation and Antimicrobial Resistance of Bovine Mastitis-Causing Bacteria in Ireland
Source: Antibiotics (Basel). 2025 Feb 27;14(3):243. doi: 10.3390/antibiotics14030243 (PMC11939333; doi:10.3390/antibiotics14030243)

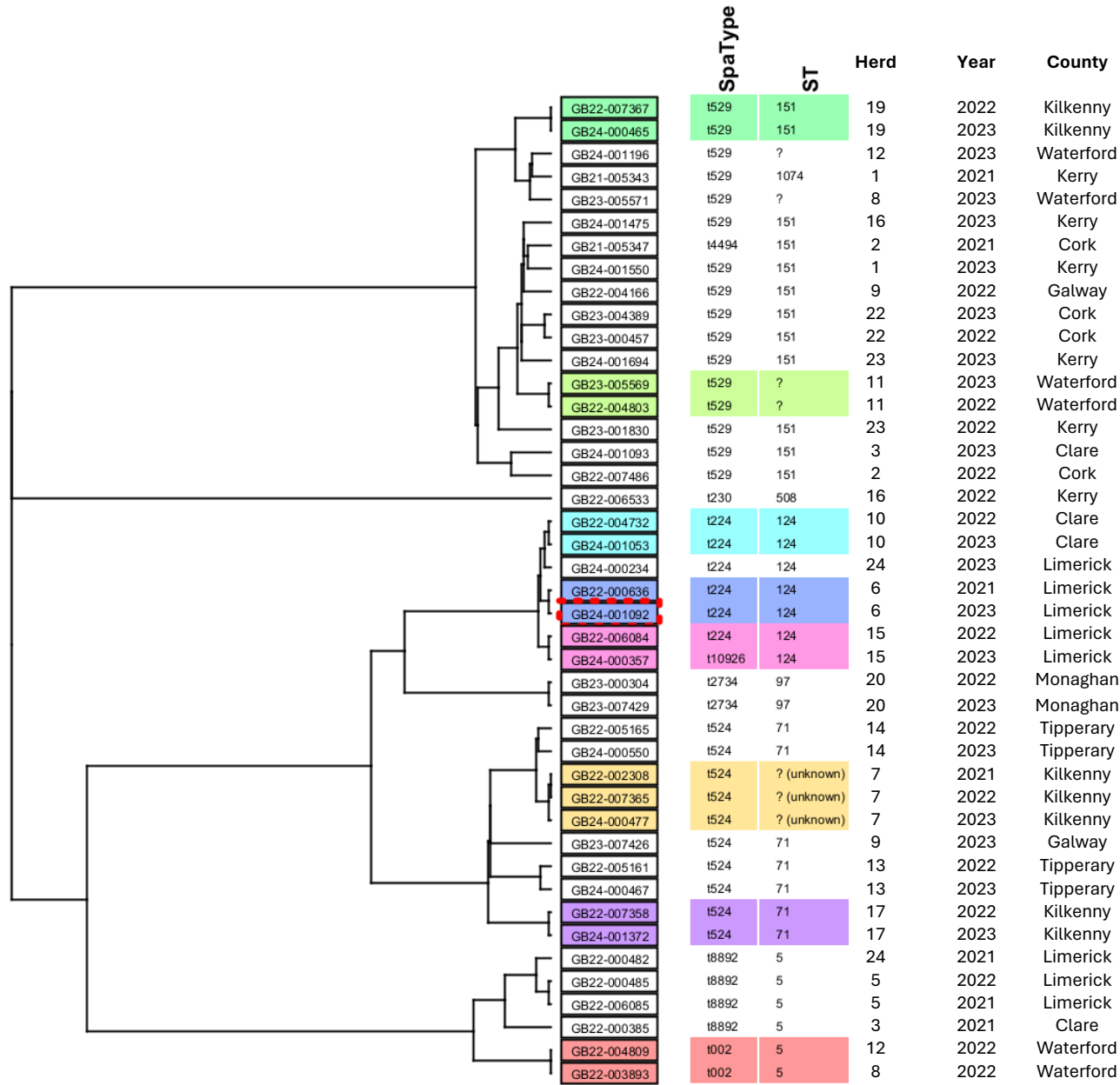

0.1

Supplement: Supplementary file 1 [file antibiotics-14-00243-s001.zip › Figure S1.pdf]
